# Overcoming Health Promotion Barriers in Rural Health: A Qualitative Analysis

**DOI:** 10.1093/geroni/igab046.492

**Published:** 2021-12-17

**Authors:** John Batsis

**Affiliations:** University of North Carolina at Chapel Hill, Chapel Hill, North Carolina, United States

## Abstract

Weight loss interventions are fraught with difficulties for older adults in rural areas due to transportation difficulties, reduced availability of staff, and lack of programs. Telemedicine can overcome these barriers. A qualitative analysis of data from 44 exit-interviews from a rural-based, older adult weight loss study, informed by thematic analysis, was conducted. Participant’s age was 73 years (73% female) and BMI was 36.5kg/m2. Distance to the site was 24 miles (31 min). Key themes included: a) telemedicine can help improve one’s health, is more practical than in-person visits, is less costly, and time efficient; b) the majority (60%) were initially apprehensive about using telemedicine, a fear that resolved quickly; c) setting up telemedicine was easy and acceptable, despite a quick learning curve; d) having a team member for troubleshooting was important. Using telemedicine in older adults with obesity residing in rural areas should be considered in health promotion interventions.

